# Association mapping for kernel phytosterol content in almond

**DOI:** 10.3389/fpls.2015.00530

**Published:** 2015-07-09

**Authors:** Carolina Font i Forcada, Leonardo Velasco, Rafel Socias i Company, Ángel Fernández i Martí

**Affiliations:** ^1^Genome Center, University of California, DavisDavis, CA, USA; ^2^Instituto de Agricultura Sostenible, Consejo Superior Investigaciones CientíficasCórdoba, Spain; ^3^Unidad de Hortofruticultura, Centro de Investigación y Tecnología Agroalimentaria de AragónZaragoza, Spain

**Keywords:** *Prunus amygdalus*, genetic variability, sterol content, population structure, linkage disequilibrium, SSR markers, candidate genes

## Abstract

Almond kernels are a rich source of phytosterols, which are important compounds for human nutrition. The genetic control of phytosterol content has not yet been documented in almond. Association mapping (AM), also known as linkage disequilibrium (LD), was applied to an almond germplasm collection in order to provide new insight into the genetic control of total and individual sterol contents in kernels. Population structure analysis grouped the accessions into two principal groups, the Mediterranean and the non-Mediterranean. There was a strong subpopulation structure with LD decaying with increasing genetic distance, resulting in lower levels of LD between more distant markers. A significant impact of population structure on LD in the almond cultivar groups was observed. The mean *r^2^*-value for all intra-chromosomal loci pairs was 0.040, whereas, the *r^2^* for the inter-chromosomal loci pairs was 0.036. For analysis of association between the markers and phenotypic traits five models were tested. The mixed linear model (MLM) approach using co-ancestry values from population structure and kinship estimates (K model) as covariates identified a maximum of 13 significant associations. Most of the associations found appeared to map within the interval where many candidate genes involved in the sterol biosynthesis pathway are predicted in the peach genome. These findings provide a valuable foundation for quality gene identification and molecular marker assisted breeding in almond.

## Introduction

Almond is the most important source of nut tree oils worldwide and is well ranked among the oil crops (http://faostat.fao.org). Kernel quality has become an important criterion for selecting modern almond cultivars (Socias i Company et al., 2008). Almond breeding has been until recently focused on selecting self-compatible and late-blooming cultivars with fruits of a high physical quality (Socias i Company et al., 2012). Consequently, very little information on the chemical evaluation of the almond kernel has been reported and the studies on the chemical components of the almond kernel and their variability are scarce (Socias i Company et al., 2008). Incorporation of such parameters in the evaluation of new crosses would be of special relevance in determining the possible commercial and industrial uses of the kernels, since their specific use depends primarily on its chemical composition (Socias i Company et al., 2008). Among the chemical parameters evaluated in recent years, the most important have been fatty acid composition (Kodad et al., 2011), tocopherol content (Kodad et al., 2006), and phytosterol content (Fernández-Cuesta et al., 2012a).

In fruit trees, classical breeding takes a lot of time and effort, especially for field management and observations of field trials (Socias i Company, 1998). The development of DNA-based technologies enables breeders to target specific traits much more effectively than using classical breeding techniques.

Phytosterols or plant sterols are products of the isoprenoid biosynthetic pathway naturally present in plants and occurring exclusively in the cytoplasm. They resemble mammalian cholesterol, both in their chemical structure and their biological function (Piironen et al., 2000). The role of phytosterols in plant growth and developmental processes such as cell division (Lindsey et al., 2003), embryogenesis (Clouse, 2000), anti-inflammatory (Bouic, 2001), and anti-oxidation activities (van Rensburget et al., 2000) is well known. Indeed, plant sterols reduce intestinal cholesterol absorption and, subsequently, LDL cholesterol (Plat and Mensink, 2005), which has been recognized since two decades ago as one of the main risk factors of cardio-vascular diseases, the leading cause of mortality in western countries (Castelli, 1984). Since plant sterols are structurally related to cholesterol and are incorporated into the mixed micelles in the intestinal tract, they are largely recommended to be included routinely in the human diet as they are one of the main mechanisms for cholesterol reduction (Law, 2000). In addition, phytosterol supplementation in humans can decrease significantly the risk of chronic diseases such as cardio-vascular disease, cancer or neurological disorders (Bramley et al., 2000). In almond oil, total phytosterol content represents the sum of the major components such as β-sitosterol (±72%) and Δ^5^-avenasterol (±15%). Other components represented in minor amounts are campesterol (±2.5%), stigmasterol (±0.8%), Δ^7^-campesterol (±3%), clerosterol (±1%), sitostanol (± 0.5%), Δ^5, 24^-stigmastadienol (±0.6%), Δ^7^-stigmastenol (±1.5%), and Δ^7^-avenasterol (±1.8%) (Fernández-Cuesta et al., 2012a).

The CITA almond germplasm collection shows a very large variability reflecting the wide genetic diversity of its accessions (≈250 accessions) from all over the world (Espiau et al., 2002). Taking this variability into account, this collection is used as the almond reference collection for the Spanish Plant Genetic Resources Network, the Spanish and European Community Plant Variety Offices. This collection has also been the origin of the CITA almond breeding program, where not only very important agronomical traits, such as late blooming and self-incompatibility, are considered, but also all aspects of almond quality (Socias i Company et al., 2012).

Molecular markers have been used extensively in almond breeding and genetic studies, and several linkage maps have been constructed for specific almond crosses to enhance efficiency of almond breeding programs (Sánchez-Pérez et al., 2007; Font i Forcada et al., 2012; Fernández i Martí et al., 2013). Linkage maps can be useful in identifying quantitative trait loci (QTL) for specific crosses and can be used for studying various traits including fruit quality, diseases resistance and physiological characteristics, among others.

As an alternative to analysis in controlled crosses, association mapping (AM) in unstructured and complex populations is now being largely applied to many crops. This approach relies on the strength of association between genetic markers and phenotype. Thus, it detects and locates genes relative to an existing map of genetic markers (Mackay and Powell, 2007). AM has been successfully applied in mapping genes involved in several traits in different plant species such as, maize (Krill et al., 2010), lettuce (Simko et al., 2009), potato (Pajerowska-Mukhtar et al., 2009), wheat (Breseghello and Sorrells, 2006), but only few studies have been carried out in fruit tree crops, such as peach (Font i Forcada et al., 2013), apple (Cevik et al., 2010), or pear (Oraguzie et al., 2010).

One of the key goals of AM is to detect marker-QTL linkage associations using plant materials routinely developed in breeding programs and that can be deployed through marker-assisted selection (MAS) in subsequent generations of cultivar development. However, AM reduces those limitations and does not require the construction of mapping populations and is performed with germplasm collections or non-structured populations. Additionally, phenotypic information available in germplasm collections might directly been used in linkage disequilibrium (LD) mapping avoiding the time required to produce F_1_ populations, especially in fruit trees. In general, AM is more suited for organisms with little or no pedigree information, populations with rich allelic diversity and traits with little or no selection history and controlled by many loci with small effects (Oraguzie et al., 2007). All these conditions are common in vegetatively propagated fruit trees, such as almond, with a high level of heterosis and a short breeding history (Socias i Company et al., 2012).

Consequently, the objective of the present study has been to perform the first whole genome AM analysis for phytosterol content in a group of 71 almond accessions to identify the genomic regions associated to this quality trait.

## Materials and methods

### Plant material and DNA isolation

A collection of 71 almond (*Prunus amygdalus* Batsch) cultivars encompassing a wide range of geographic origins was used in this study (Table 1). They were selected among the whole almond pool in order to have the most representative Spanish local accessions, including 36 genotypes from all the Spanish growing regions. In addition, cultivars from different breeding programs and some foreign cultivars (USA, France, Greece, Italy, Portugal, Algeria, Argentina, Australia, Bulgaria, Tunisia and Ukraine) were also included in this study. The trees are maintained as living plants grafted on the almond × peach hybrid clonal rootstock INRA GF-677, using standard management practices (Espiau et al., 2002).

**Table 1:** **Almond accessions studied, and the country and region of origin**.

**Cultivar**	**Origin**	**Cultivar**	**Origin**	**Cultivar**	**Origin**
Alcina	Spain	Malagueña	Spain	Texas	United States
Aspe	Spain	Marcona	Spain	Thompson	United States
Atocha	Spain	Marcona Argentina	Argentina	Tioga	United States
Belle d'Aurons	France	Mardía	Spain	Tokyo	United States
Blanquerna	Spain	Marta	Spain	Torreta	Spain
Cambra	Spain	Masbovera	Spain	Truito	Greece
Carreirinha	Portugal	Menut	Spain	Tsotouliu	Greece
Cartayera	Spain	Molar de Fuzeta	Portugal	Tuono	Italy
Castilla	Spain	Mono	United States	Vinagrilla	Spain
Chellastone	Australia	Muel	Spain	Vivot	Spain
Colorada	Spain	Padre Santo	Spain	Yaltinskij	Ukraine
Constantini	Algeria	Pané-Barquets	Spain	Zahaf	Tunisia
Cosa Nova	Portugal	Peerless	United States	Zinia	Spain
Desmayo Largueta	Spain	Phyllis	Greece		
Desmayo Rojo	Spain	Picantilli	Greece		
Dura de Tijarafe	Spain	Primorskij	Ukraine		
El Paso-4	Spain	Rachele	Italy		
Emilito	Argentina	Rameira	Portugal		
Exinograd	Bulgaria	Raposa	Portugal		
Ferraduel	France	Redonda de Palma	Spain		
Ferragnès	France	Rof	Spain		
FilippoCeo	Italy	Sovietskij	Ukraine		
Forastero	Spain	Supernova	Italy		
Garfi	Spain	Symmetrikji	Greece		
Garondès	Spain	Taiatona	Spain		
Garrigues	Spain	Tardy Nonpareil	United States		
Glorieta	Spain	Tarragonès	Spain		
I.X.L.	United States	Tejeda 1	Spain		
Lauranne	France	Tendra Amarga	Spain		

Twenty mature fruits were randomly collected from each genotype during two consecutive years. The fruit was considered mature when the mesocarp was fully dry and split along the fruit suture and the peduncle was near to complete abscission. Fruits were cracked and seed coats removed by pouring in warm water (100°C) during 5 min. Blanched kernels were dried until constant weight and ground in an electrical grinder to obtain fine flour. Almond oil was extracted from the almond flour to analyze the phytosterol content (Fernández-Cuesta et al., 2012a).

For DNA extraction, leaf samples were collected from young shoots from the upper part of each tree, frozen immediately in liquid nitrogen, and stored at -20°C. Genomic DNA was isolated following the PowerPlant DNA isolation Kit (MO BIO Laboratories, CA, USA). The DNA was quantified and diluted to 10 ng uL-1 for PCR amplifications.

### Phenotypic evaluation

Phytosterol content was analyzed in two replicates per sample following a previously described procedure for the analysis of free and esterified phytosterols (Fernández-Cuesta et al., 2012a). In short, almond flour to which an internal standard solution of cholesterol 0.1% was added to alkaline hydrolysis with potassium hydroxide 2% followed by phytosterols extraction in hexane:water (1:1.5 by vol.). Phyosterols were then derivatized for 15 min at room temperature using a commercial silylating mixture (Silan-Sterol-1, Panreac Química, Barcelona, Spain) and analyzed by gas chromatography using the analytical conditions reported by Fernández-Cuesta et al. (2012b). Kernel phytosterol content was expressed as milligrams per kilogram of kernel. Theoretical oil phytosterol content, expressed as milligrams per kilogram of kernel oil, was estimated from kernel phytosterol content and kernel oil content using the following formula: *oil phytosterol content = (kernel phytosterol content × 100)/oil content*.

### Genotyping

Forty fluorescently labeled microsatellite (SSR) markers (Table 2) developed in other *Prunus* species were used to genotype the 71 accessions. These SSR have been chosen because of their high polymorphism in peach, cherry, plum, and almond, and because they represent a wide coverage of the almond genome. PCR conditions were as follow: 1×PCR buffer, 1.5 mM MgCl_2_, 0.2 mM dNTPs, 0.2 μM of each primer, one unit of Taq DNA Polymerase (Invitrogen, Madrid, Spain) and 20 ng of genomic DNA in a 20 μl final volume. The PCR program consisted in a denaturation for 1 min at 94°C, followed by 35 cycles of 15 s at 94°C, 15 s for the annealing temperatures for the different primers used, and 1 min at 72°C, and a final extension of 2 min at 72°C. Each reaction was repeated and analyzed twice to ensure reproducibility. PCR products were detected using ABI 3130xl Genetic Analyzer and GeneMapper (Applied Biosystems). For capillary electrophoresis detection, forward primers were labeled with 5′-fluorescence dyes PET, NED, VIC and 6-FAM and the size standard was Gene Scan™ 500 Liz® (Applied Biosystems).

**Table 2:** **Genetic parameters of 71 almond cultivars based on 40 SSR loci**.

**SSR**	***A***	***Ae***	***Ho***	***He***	***Fis***	***I***	***PD***
BPPCT011	19	7.7	0.78	0.87	0.10	23.13	0.87
CPPCT053	23	12.5	0.83	0.92	0.09	27.12	0.92
EPDCU5100	2	1.3	0.25	0.22	−0.14	0.37	0.18
BPPCT001	15	4.5	0.39	0.78	0.50	19.95	0.79
BPPCT030	4	1.3	0.24	0.22	−0.12	0.44	0.20
CPPCT044	18	4.8	0.70	0.79	0.11	20.79	0.69
CPSCT021	14	4.8	0.69	0.79	0.13	19.45	0.92
PceGA34	13	2.4	0.56	0.58	0.03	13.77	0.61
BPPCT007	16	8.3	0.83	0.88	0.05	23.27	0.88
BPPCT039	15	11.1	0.67	0.91	0.24	24.58	0.90
CPDCT025	21	11.1	0.81	0.91	0.11	26.47	0.93
EPDCU0532	9	4.8	0.78	0.79	0.01	17.14	0.79
UDP96-008	5	2.2	0.53	0.55	0.03	0.97	0.43
BPPCT010	16	7.1	0.41	0.86	0.52	22.42	0.93
CPDCT045	17	10.0	0.90	0.90	−0.02	24.89	0.92
CPPCT005	20	10.0	0.87	0.90	0.03	25.27	0.95
EPPCU6216	16	5.9	0.75	0.83	0.09	21.37	0.84
EPPCU9168	12	4.3	0.75	0.77	0.02	19.07	0.77
PMS40	18	5.3	0.75	0.81	0.07	20.72	0.81
PS12e2	16	8.3	0.85	0.88	0.03	23.40	0.89
UDP96-003	21	10.0	0.76	0.90	0.14	24.93	0.93
UDP97-401	17	9.1	0.67	0.89	0.24	24.04	0.90
BPPCT038	11	5.6	0.78	0.82	0.03	19.25	0.81
CPPCT009	9	3.2	0.57	0.69	0.16	16.01	0.68
CPPCT040	20	12.5	0.94	0.92	−0.03	26.69	0.92
CPSCT006	7	2.6	0.64	0.62	−0.03	11.82	0.62
CPSCT022	8	1.9	0.42	0.48	0.13	0.96	0.47
PceGA25	18	6.2	0.75	0.84	0.11	21.73	0.84
BPPCT025	16	8.3	0.78	0.88	0.11	23.07	0.90
CPPCT008	7	4.0	0.71	0.75	0.04	15.12	0.72
CPPCT021	17	3.1	0.42	0.68	0.38	16.61	0.68
CPPCT047	19	6.7	0.71	0.85	0.17	23.34	0.90
CPSCT012	16	10.0	0.72	0.90	0.20	24.60	0.90
MA040	10	4.8	0.68	0.79	0.13	18.18	0.79
EPDCU3392	10	5.6	0.48	0.82	0.42	19.33	0.82
CPPCT022	19	5.3	0.59	0.81	0.27	21.23	0.81
EPPCU7340	16	8.3	0.67	0.88	0.24	22.82	0.88
PMS02	4	1.6	0.32	0.39	0.18	0.79	0.35
CPPCT006	21	11.1	0.92	0.91	−0.02	26.35	0.96
CPSCT018	2	1.7	0.55	0.41	−0.38	0.59	0.40
Mean	13.9	4.2	0.66	0.76	0.11	18.20	0.76

*A, observed number of alleles per locus; A_e_, effective number of alleles per locus; H_o_, observed heterozygosity; H_e_, expected heterozygosity; F_is_, Wright's fixation index; I, Shannon's information index; PD, power of discrimination*.

### Phenotype-genotype association analysis

Genetic parameters such as the number of alleles per locus (*A*), the effective number of alleles detected per locus (*A_e_*), the observed heterozygosity (*H_o_*), the expected heterozygosity (*H_e_*), and the Wright's fixation index (*F*) were obtained for comparing both heterozygozities. All parameters were estimated using the PopGene 1.31 software. Phylogenies were estimated using Neighbor joining algorithm/method (Saitou and Nei, 1987). Neighbor joining analyses were conducted with the PAUP^*^ v.4.0b10 phylogenetics package (Swofford, 2003).

Population structure in our diverse collection of almond cultivars was addressed using the method based on the Bayesian modeling environment implemented in the software STRUCTURE 2.3.2 (Pritchard et al., 2000). The aim of this approach was the identification of population structure by clustering individuals into genetically distinguishable groups on the basis of allele frequencies. The *ad hoc* statistic Δ*K* (Evanno et al., 2005) was used to set the number of populations (*K*). Individual and admixture analyses were performed using STRUCTURE assuming an admixture model where the allelic frequencies were correlated. This method uses a Markov Chain Monte Carlo (MCMC) algorithm to cluster individuals into populations on the basis of multilocus genotype data (Pritchard et al., 2000). A burn-in of 20,000 and 250,000 MCMC replications seemed to be the best fit for our data at *K* = 3. The analysis was run for *K*-values ranging from two to ten inferred clusters with 20 independent runs each. The results were displayed graphically in a bar graph/chart. The low frequency alleles (considering *MAF* ≤ 0.05) were removed. The LD between pairs of multiallelic loci was calculated separately for loci on the same or on a different linkage group (LG), using the *r^2^* coefficient. The statistical *r^2^* gives an indication of both recombination and mutation (Flint-Garcia et al., 2003). The significance level of LD between loci was examined using a permutation test implemented in TASSEL software for multiallelic loci, using the “rapid permutation” option.

AM analyses were measured by using TASSEL 2.1 (Yu and Buckler, 2006). The General Linear Model (GLM, Q) and the Mixed Linear Model (MLM, Q + K) approaches were used to examine association between the phenotypic traits and DNA markers. A structured association approach could correct false associations using Q-matrix of population membership estimates. The mean value of the markers at *P* < 0.005 was used for determining the significance of marker-trait associations. Significant markers were declared using the Bonferroni procedure at the *p* < 0.00125 experimental-wide threshold. Alleles with minor frequency (MAF) lower than 5% were removed (Wilson et al., 2004).

## Results

### Phenotypic variation in the almond germplasm

The phenotypic variability of the phytosterol content and profile was large, reflecting the wide coverage of the almond gene pool reached by the 71 cultivars studied. The distribution for total phytosterol content was left-skewed, with a range from 2776.5 to 1125.6 mg kg^−1^ and a mean of 1882.5 mg kg^−1^. Most phytosterol content traits showed a normal distribution when evaluating their frequency, indicating that this subset was not biased (Table 3). These results remark the importance of the genetic background of each accession for the phenotypic profile of its nuts.

**Table 3:** **Units, minimum, maximum, and mean values for the phytosterol traits evaluated in 71 almond cultivars (average of 2 years of study)**.

**Trait**	**Units**	**Maximum**	**Minimum**	**Mean ± SE**
Total phytosterol	(mg kg^−1^)	2776.50	1125.60	1882.5 ± 303.1
Oil	%	69.20	47.24	60.3 ± 2.93
Oil phytosterol content	(mg kg^−1^)	4553.90	1897.91	3120.1 ± 459.9
Campesterol	%	6.10	1.39	2.74 ± 0.68
Stigmasterol	%	2.90	0.18	0.73 ± 0.40
Δ^7^-Campesterol	%	9.82	0.13	2.86 ± 1.52
Clerosterol	%	2.75	0.41	1.26 ± 0.35
β-Sitosterol	%	84.60	55.90	73.1 ± 4.24
Δ^5^-Avenasterol	%	28.20	8.45	15.5 ± 3.11
Δ^7^-Stigmastenol	%	4.82	0.11	1.24 ± 0.90
Δ^7^-Avenasterol	%	4.63	0.24	1.46 ± 0.79

### SSR analysis of the accessions

Amplification of the 40 SSR loci was successful in all the almond genotypes, producing well-defined and reproducible bands. The primers produced a total of 501 different alleles, with an average of 13.9 alleles per locus, with sizes from 86 to 302 bp. The marker EPDCU5100 showed only two alleles whereas the marker CPPCT053 produced 23 different alleles (Table 2). All primers produced a maximum of two bands per genotype, according to the diploid level of almond. The observed heterozygosity ranged from 0.24 (BPPCT030) to 0.94 (CPPCT040), with an average of 0.66 across all 40 SSRs. H_o_ and H_e_ values were compared with the fixation index (F) which was on the average 0.11, ranging from −0.14 (EPDCU5100) to 0.52 (BPPCT010). The high *F*-values observed corresponding to high homozygosity, particularly in individuals with only one band, suggests the presence of null alleles (Brookfield, 1996). The *F*-value was positive in 33 and negative in 7 SSR loci, thus indicating the high level of heterozygosis in the cultivars studied, as it would be expected in an allogamous species such as almond. It was shown that the SSRs developed in other *Prunus* species such as peach, sweet cherry or Japanese plum, can be effectively used for evolutionary and fingerprinting studies in almond, confirming the high level of synteny within the *Prunus* species (Mnejja et al., 2010).

### Clustering analysis of the genotypes

**Figure 1:**
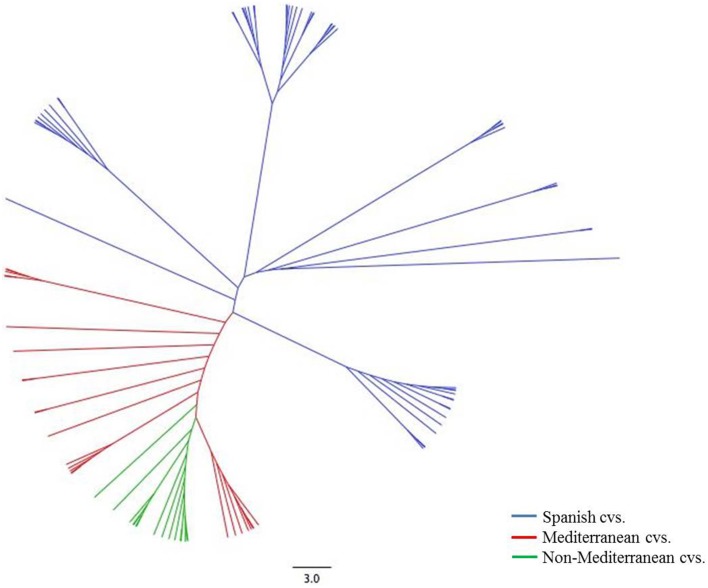
**Neighbor joining tree of 71 almond accessions based on 40 SSR markers**.

Clustering analysis based on Neighbor-joining essentially allowed the detection of three major clusters of different size, further subdivided in other small clusters (Figure 1). A close relationship could be established for some of these groups with their geographical origin. The first cluster (blue) contained only Spanish accessions (36 in total). This group comprises only cultivars from Spain with representative accessions such as “Marcona,” “Desmayo Largueta,” “Atocha” or “Castilla.” Some new releases from different Spanish breeding programs, such as “Mardía” and “Marta,” were also grouped in this cluster. The accessions from the two Spanish archipelagos (Canary Islands and Majorca), such as “Padre Santo,” “Dura de Tijarafe,” “Vivot,” “Garondès” and “Vinagrilla,” were equally clustered in this first group. The second group (red) appeared to be much diversified, with 20 accessions, including a pool of Mediterranean accessions: two from North Africa (“Constantini” and “Zahaf”), four from France (“Belle d'Aurons,” “Lauranne,” “Ferragnès,” and “Ferraduel”), four from Italy (“Tuono,” “Supernova,” “Rachele,” and “Filippo Ceo”), five from Greece (“Picantilli,” “Tsotouliu,” “Truito,” “Symmetrikji,” and “Phyllis”), and five from Portugal (“Cosa Nova,” “Molar de Fuzeta,” “Carreirinha,” “Raposa,” and “Rameira”). The third group (green) clustered accessions from many different countries, although all of them have in common not being strictly Mediterranean. This group included one accession from Bulgaria (“Exinograd”), three from Crimea (“Yaltinskij,” “Primorskij,” and “Sovietskij”), two from Argentina (“Marcona Argentina” and “Emilito”), one from Australia (“Chellastone”) and eight from the United States (“I.X.L.,” “Texas,” “Thompson,” “Tioga,” “Peerless,” “Tokyo,” “Mono,” and “Tardy Nonpareil”).

### Population structure and linkage disequilibrium analysis

**Figure 2:**
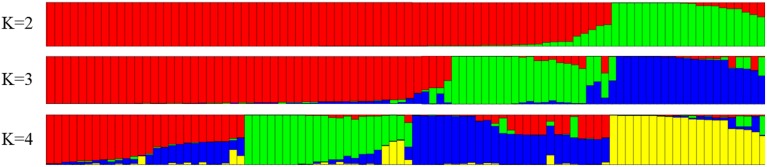
**Structure clustering for 71 almond accessions using 40 SSR**.

The co-dominant nature of the 40 molecular markers was used to analyze the structure of the populations using a Bayesian approach. The number of subpopulations (*K*) tested ranged between 2 and 10 (Figure 2) with 20 runs for each *K* using MCMC replications which showed evident knees at *K* = 3 (Evanno et al., 2005). The level of partitioning corresponded to a very strong differentiation into two major groups. The first group contained accessions from Mediterranean countries, mostly from Spain, but also from Italy, France, Portugal, Greece and North Africa, while the second group contained accessions from non-Mediterranean countries (including America, Australia and Eastern Europe). The proportion of genotypes assigned to each population was not symmetric, indicating that population structure exists (Pritchard et al., 2000).

**Figure 3:**
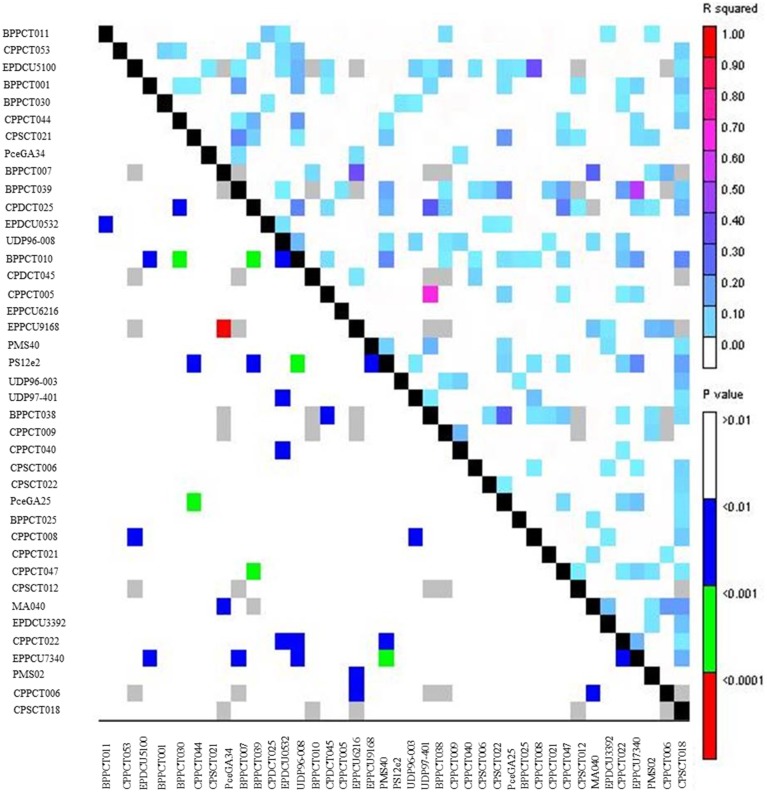
**LG plot based on 40 SSR screened on 71 almond accessions**.

**Figure 4:**
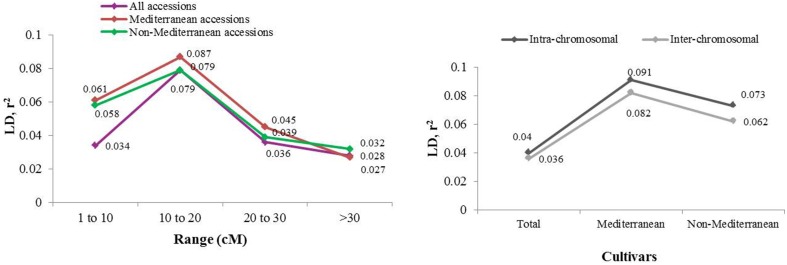
**LD based on *r^2^*, averaged for map distance classes and germplasm groups based on population structure analysis in the STRUCTURE**.

Extent of genome-wide LD was evaluated through pairwise comparisons among the 40 marker loci and the 71 almond germplasm accessions studied (Figures 3, 4). After removing low frequency alleles (*MAF* = 0.05), the results showed a high level of LD up to 20 cM, which dissipated at farther distances. The overall LD for all cultivars was 0.034 in the region from 0 to 10 cM, 0.079 from 10 to 20 cM, 0.036 from 20 to 30 cM, and 0.027 after 30 cM. These results were lower if the cultivars were separated in two different groups, Mediterranean and non-Mediterranean. Thus, the range of LD spaced every 10 cM was 0.061, 0.087, 0.045, and 0.032 for Mediterranean cultivars, and 0.058, 0.079, 0.039, and 0.028 for non-Mediterranean cultivars. A high level of LD up to 20 cM was observed for the whole ensemble of accessions when they were split in a Mediterranean and a non-Mediterranean group. The total *r^2^*-value for intra-chromosomal loci pairs was 0.040 and the unlinked markers pairs showed a similar percentage of significant LD in Mediterranean and non-Mediterranean cultivars (values of 0.091 and 0.073, respectively). Regarding the total *r^2^*-value for inter-chromosomal loci pairs was 0.036 for the whole ensemble, 0.082 for the Mediterranean accessions, and 0.062 for the non-Mediterranean accessions. The overall level of LD detected was low, which could be mostly likely due to poor marker coverage.

### Association mapping and allelic effect

We tested five models in TASSEL to determine associations and also to account for the influence of population structure by comparing their ability to reduce the inflation of false positive associations. The *P*-values were plotted in a cumulative fashion for each model and the distribution examined. According to Stich et al. (2008) the distribution of *P*-values ideally should follow a uniform distribution with less deviation from the expected *P*-values.

The association analysis using the GLM approach (being the naïve model), Q-model and P-model, detected a large number of associations between the markers and phenotypes. The Q-model (GLM with Q-matrix as correction for population structure) showed 30 associations between markers and traits (results not shown). It appears that these models may not have accounted for the heterogeneity of the genetic background, which may have resulted in false positive associations.

**Figure 5:**
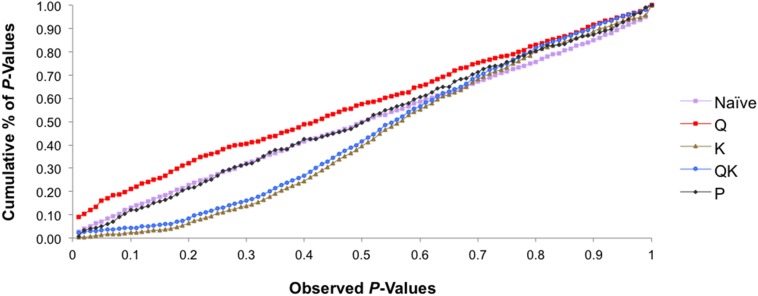
**Comparison of different genome wide association study (GWAS) models**. Cumulative distribution of *P*-values was computed from the DNA markers and phenotypes for the different association models.

The K-model (MLM with K-matrix as correction for population structure) and QK-model (MLM with Q-matrix and K-matrix as correction for population structure) showed good fit for the *P*-values (*P* < 0.001), while the other models were characterized by the excess of small *P*-values (abundance of spurious associations) (Figure 5). These two latter models showed high uniform distribution of *P*-values.

Taking into account the performance of the different models, only results from the K-model are showed and discussed here since this appeared to have controlled better population structure and kinship relationships. Thirteen significant associations were detected between seven SSRs and eight phytosterol traits (Table 4). The BPPCT011 locus was associated with total phytosterol, stigmasterol and Δ^7^-stigmastenol contents. The EPDCU5100 and BPPCT030 markers were associated with β-sitosterol and stigmasterol, respectively. The CPPCT008 and UDP96-003 markers were associated with clerosterol and Δ^7^-stigmastenol and with total phytosterol and Δ^7^-avenasterol, respectively. Finally, the markers CPPCT047 and EPDCU3392 were associated with Δ^7^-campesterol and Δ^7^-stigmastenol, and with campesterol and Δ^7^-stigmastenol, respectively. The percentage of phenotypic variation explained by these markers ranged between 73.8 and 46.1%, with UDP96-003 having the maximum value and EPDCU5100 the minimum value. The *P*-values showing the level of significance of the associations between SSR markers and phytosterol traits are shown in Figure 5. Some associations were observed in the same regions where QTL had previously been identified (Table 4).

**Table 4:** **Statistical significance of the** ***p*****-values and associations observed between markers and phytosterol content of almond cultivars**.

	**BPPCT011^a^**	**EPDCU5100**	**BPPCT030**	**CPPCT008**	**UDP96-003^a^**	**CPPCT047**	**EPDCU3392^a^**
SSR/LG	1	1	2	6	4	6	7
% var (*R*^2^)^b^	62.6	46.1	56.8	66.7	73.8	51.8	50.0
Total phytosterol	^***^				^**^		
Campesterol							^**^
Stigmasterol	^*^		^**^				
Δ^7^-Campesterol						^*^	
Clerosterol				^***^			
β-Sitosterol		^**^					
Δ^7^-Stigmastenol	^**^			^**^		^**^	^***^
Δ^7^-Avenasterol					^**^		

For multiple testing of genotypes, Bonferroni correction (Schulze and McMahon, 2002) was applied. The p-values for associations are considered when at least one allele is associated with the SSR. Abbreviations: W, width; T, thickness; L, length

* *p < 0.00001*,

** *p = 0.00001–0.0001*,

*** *p = 0.0001–0.0012, (K-model)*.

a *Associations observed in the same regions where QTL had previously been identified (Font i Forcada et al., 2012; Fernández i Martí et al., 2013)*.

b *Percentage of the phenotypic variation (r^2^) explained by each marker*.

## Discussion

Phytosterol content in plants is a complex trait known to be controlled by both major and minor genetic factors (Amar et al., 2008). As far as we know, this is the first time that the phytosterol content is genetically studied in almond, and also it represents the second AM analysis in this nut crop (Font i Forcada et al., 2015). However, there have so far been few studies on genotypic effects and QTL mapping phytosterols in other species. The few studies that have been documented are in sunflower (Haddadi et al., 2012; Merah et al., 2012) and *Brassica napus* L. (Amar et al., 2008; Shia, 2014).

The mean allele number found in this study, 13.9, was slightly lower than 17.2 obtained by Fernández i Martí et al. (2009), but very similar to 14.6 reported by Elhamzaoui et al. (2012), and much higher than 4.7 obtained by Martínez-Gómez et al. (2003). One possible reason for this discrepancy in the mean number of alleles (17.2 vs. 13.9) could be the inclusion of wild genotypes by Fernández i Martí et al. (2009), resulting in more alleles, some of which novel, whereas only domesticated germplasm was used in the present study, possibly having narrowed their genetic base. The average heterozygosity value of 0.66 is lower than 0.72 obtained by Fernández i Martí et al. (2009), but slightly higher than other values reported in almond, 0.62 by Elhamzaoui et al. (2012) and 0.59 by Martínez-Gómez et al. (2003).

The two main genetic groups identified by the Bayesian analysis corresponded to the Mediterranean and non-Mediterranean gene pools. These two groups could also be further substructured based on geographical origin. These clusters, however, could also be associated with local adaptation, diversifying selection, familial relatedness, or combinations thereof (Yu et al., 2006). Many species have undergone a long and complex period of domestication and breeding with limited gene flow, and this could be expected in the structure of this complex population (Sharbel et al., 2000). Hence, despite the diversity observed in almond, genetic bottlenecks may have occurred during almond dissemination (Fernández i Martí et al., 2015). The presence of population stratification and unequal distribution of alleles could result in non-functional, spurious associations (Pritchard and Rosenberg, 1999). In order to understand the distribution of genetic diversity in the almond cultivars, the model-based clustering approach was implemented in STRUCTURE to infer population structure. Thus, the 71 accessions could be separated in two different pools, the Mediterranean and non-Mediterranean, which are in line with those obtained by Fernández i Martí et al. (2015), where only 17 SSRs were used. In addition, in that study the cultivars from Europe also grouped separately from the Asian, American and Australian cultivars.

LD depends on a combination of many factors, such as the origin of the population, the selected set of accessions, the analyzed genomic region, the molecular marker system, and the presence of unidentified subpopulations. However, our LD results are very similar to those obtained by Fernández i Martí et al. (2015) (*r*^2^ = 0.04 for intra-chromosomal and *r*^2^ = 0.03 for inter-chromosomal), as well as in another self-incompatible *Prunus* species, such as sweet cherry (*r*^2^ = 0.028) (Arunyawat et al., 2012). Although LD in general decays more rapidly in out-crossing species than in selfing species, since recombination may be less effective in selfing species (Nordborg and Tabare, 2002), the level of LD decay observed in our study is comparable to the decay found in a self-compatible *Prunus* species such as peach (Cao et al., 2012; Font i Forcada et al., 2013). Population structure influences the magnitude and pattern of LD in almond, as in other species, such as *Arabidopsis thaliana* (Ostrowski et al., 2006), maize (Remington et al., 2001), and barley (Comadran et al., 2009). In the presence of LD, it will be possible to identify genetic regions (if LD extends to a distance of several cM) or genes (if LD decays quickly, in a few thousand base pairs) associated with a particular trait of interest by genome-wide associations or by individual SNPs (Single Nucleotide Polymorphisms) or SNP haplotypes within a candidate gene (Malysheva-Otto et al., 2006), respectively.

A previous study (Fernández i Martí et al., 2015) showed a strong subpopulation structure and LD decaying with increasing genetic linkage distance using only 17 SSR. Although the number of markers used in the present study was relatively low (40 SSR), our associations represent a first attempt of identification of candidate genes and provide strong evidence for the AM of phytosterol content in almond. Correction for the confounding effects of population structure present in plant populations is essential for AM because the complex population structure may cause spurious correlations (Pritchard et al., 2000). In our almond germplasm, we tested five models in TASSEL, and among them, K-model seems to have controlled population structure and kinship relationships better to eliminate the possible spurious associations. Results from this analysis have been corrected to minimize spurious associations leading to less inflated type I error (Yu et al., 2006).

Among the 40 SSR loci associated with phytosterol traits by association analysis (13 significant associations), many markers previously identified by linkage mapping were included (Font i Forcada et al., 2012). For example, out of the eight SSR markers identified for phytosterol traits in this study, three of them (BPPCT011, UDP96-003, and EPDCU3392) were significantly associated (seven significant associations) or located near regions where QTL had previously been identified in previous genetic studies (Font i Forcada et al., 2012). Furthermore, these seven associations were distributed among LG1, LG4, and LG7.

The complex traits detected in this study revealed that several putative genomic regions are involved in the expression of these phenotypes. To confirm the candidate genes and the marker location for the phytosterol traits, we screened the genes that belong to the plant sterol biosynthesis pathway and that are close to the associated markers found here using the browser *Prunus persica* genome sequence data (http://www.rosaceae.org). Interestingly, all the associations found in this work and located on LG1, LG2, LG4, LG6, and LG7 appeared to map within the interval where many candidate genes involved in the sterol biosynthesis pathway are predicted in the peach genome. For example, at the upper region of the LG1 and close to marker EPDCU5100, two genes (ppa022710m and ppa001844m), were identified at the same position than our EPDCU5100 marker and it might be responsible for the enzymatic reactions of cycloartenol synthase 2, SMT1.

This SSR marker was found to be associated with β-sitosterol analyzed here. Hence, the location surrounding this SSR might be considered as a good candidate region with genes controlling these phytosterols. Additionally, five more candidate genes also located on LG1 have been identified to control limiting steps in the biosynthesis of the end product sterol in plant cells (ppa012513m, ppa003821m, ppa011030m, ppa005590m, and ppa003991m). 24-Methylene sterol, cytochrome P450, Δ^24^-sterol reductase (DWF1), and squaleneepoxidase (SEQ1) appeared to be within the interval flanked by the two SSRs BPPCT011 and CPPCT053, which are associated in our study with contents of total phytosterol, and of stigmasterol and Δ^7^-stigmastenol.

Our analysis from genome scanning also found a candidate gene, ppa1027202m, strongly correlated with all the sterol traits analyzed here. This gene spans along the region where PMS40, CPDCT045 and UDP96-003 map on LG4 and it has been associated with the cytochrome P450, which encodes the sterol C-22 sterol desaturase. Finally, the results of the associations performed on LG7 were confirmed by the presence of another candidate gene, which is associated with C14 sterol reductase and ergosterol biosynthesis ERG4 (ppa007403m). This gene (scaffold 7: 14,682,911) is physically close to the SSR EPDCU3392 in the peach genome.

Almond has a poorly developed genomic infrastructure as compared to other Rosaceae species (peach, apple, strawberry, sweet cherry, etc…). With the application of new sequencing technologies, the availability of thousands of high-throughput single nucleotide polymorphisms (SNP) and various genomes of this family already sequenced (Velasco et al., 2010; Shulaev et al., 2011; Verde et al., 2012), yet relying on a set of SSR markers for identifying genes and/or QTL could be considered as a limiting factor. The application of NGS technologies and bioinformatic tools to generate high frequency SNPs still remains unexplored. Hence, the use of high-density genetic linkage maps based on SNP markers in genome mapping and phenotypic selection is still very limited in almond germplasm and breeding populations. Certainly, the use of such a small number of expressed sequence tags (EST) and microsatellite (SSR) might have somehow disadvantaged deeper conclusions in the present work, but our results match very well with most of the predicted genes found during the peach genome sequence project. Moreover, SSRs still remain very attractive for breeding purposes and they have proven superior power than other markers for genetic analysis (Khan and Korban, 2012). In addition, and as documented in the few studies on AM in *Prunus*, such as peach (Cao et al., 2012; Font i Forcada et al., 2013) and sweet cherry (Ganopoulos et al., 2011), the authors used mostly SSR markers for carrying out their findings. Marker numbers ranged in those studies from 15 SSRs in Ganopoulos et al. (2011) to 40 SSR in Font i Forcada et al. (2013) and 53 SSR in Cao et al. (2012). Thus, the 40 SSR markers used along our analysis may appear adequate for AM in almond.

It is noteworthy that an international consortium, in which our group participate, has recently been created in 2014 to sequence the whole genome of the almond cultivar “Texas” for intended release at the end of 2015 (Almond International Consortium). This advancement in genome sequencing will offer important new possibilities for SNP discovery and genome wide association studies (GWAS) in this nut crop. The cost of growing and maintaining tree crops until they reach maturity is very high, thus any effort to carry out early selection will be highly desirable to reduce orchard costs at the seedling stage.

## Conclusion

In the present study, we identified seven markers associated with phytosterol content in almond. It has also been shown that wide genetic variation exists for kernel sterol contents in this nut crop. Our results showed that AM could be highly useful to detect significant complex traits of interest. Since this is the first genetic analysis for phytosterol content in almond, we suggest that these results provide an insight into the genetic architecture of important fruit quality traits for almond and this new genetic information would offer an efficient platform for classical and MAS breeding in this *Prunus* species.

## Author contributions

CF and AF designed the study and performed the molecular and statistical analysis. LV conducted the phytosterol analysis. RS provided the material from the almond germplasm collection. CF, RS, and AF discussed the results and drafted the manuscript. All authors have read and approved the final manuscript.

### Conflict of interest statement

The authors declare that the research was conducted in the absence of any commercial or financial relationships that could be construed as a potential conflict of interest.
